# Coherent crystal branches: the impact of tetragonal symmetry on the 2D confined polymer nanostructure

**DOI:** 10.1107/S2052252521000774

**Published:** 2021-02-06

**Authors:** Ziying Liang, Nan Zheng, Bo Ni, Ziwei Lai, Hui Niu, Shuailin Zhang, Yan Cao

**Affiliations:** a Institute for Advanced Study, Shenzhen University, Guangdong 518060, People’s Republic of China; b State Key Laboratory of Luminescent Materials and Devices, South China University of Technology, Guangdong 513060, People’s Republic of China; c South China Advanced Institute for Soft Matter Science and Technology, School of Molecular Science and Engineering, South China University of Technology, Guangzhou 510640, People’s Republic of China; d Department of Polymer Science and Engineering, School of Chemical Engineering, Dalian University of Technology, Dalian 116024, People’s Republic of China; e Department of Polymer Science, The University of Akron, Akron, OH 44325, USA

**Keywords:** cylindrical confinement, coherent crystal branch, tetragonal crystal, poly(4-methyl-1-pentene)

## Abstract

The symmetry of polymer crystals greatly affects the optical, thermal con­ductivity and mechanical properties of the materials. Past studies have shown that the two-dimensional (2D) confined crystallization of polymer nanorods could produce anisotropic structures. However, few researchers have focused on understanding confined nanostructures from the perspective of crystal sym­metry. In this research, we demonstrate the molecular chain self-assembly of tetragonal crystals under cylindrical confinement. We specifically selected poly(4-methyl-1-pentene) (P4MP1) with a 4_1_ or 7_2_ helical conformation (usually crystallizing with a tetragonal lattice) as the model polymer. We found a coherent crystal branching of the tetragonal crystal in the P4MP1 nanorods. The unusual 45°- and 135°-{200} diffractions and the meridional 220 diffraction (from 45°-tilted crystals) have shown a uniform crystal branching between the *a*_1_-axis crystals and the 45°-tilted crystals in the rod long axis, which originates from a structural defect associated with tetragonal symmetry. Surprisingly, this chain packing defect in the tetragonal cell can be controlled to develop along the rod long axis in 2D confinement.

## Abstract 2

The impact of tetragonal crystal symmetry on the two-dimensional (2D) confined polymer nanostructure is demonstrated. Our research shows that this chain packing defect in the tetragonal cell can be controlled to develop along the rod long axis in 2D confinement.

## 1. Introduction   

Confinement through a nanoporous template is an effective way to produce anisotropic polymer nanostructures (Steinhart *et al.*, 2006 ▸; Higuchi *et al.*, 2012 ▸; Jinnai *et al.*, 2012 ▸; Zhu *et al.*, 2001*b* ▸; Dai *et al.*, 2018 ▸; Lai *et al.*, 2020 ▸; Yu *et al.*, 2019 ▸; Zeng *et al.*, 2019 ▸; Hsiao *et al.*, 2008 ▸; Ho *et al.*, 2005 ▸). Previous insightful works on confined polymer nanostructures are mainly related to nanophase morphologies, crystal nucleation and orientations and molecular dynamics (Cao *et al.*, 2014 ▸; Chung *et al.*, 2010 ▸; Wu *et al.*, 2012 ▸, 2013 ▸; Nakagawa *et al.*, 2012 ▸; Danch & Osoba, 2003 ▸; Lutkenhaus *et al.*, 2010 ▸; Zhu *et al.*, 2001*a* ▸; Huang *et al.*, 2001 ▸, 2006 ▸). Recently, there has been great interest in discovering the impact of the symmetry of polymer crystals under cylindrical confinement *via* nanoporous alumina under 2D confinement (Zeng *et al.*, 2019 ▸; Lai *et al.*, 2020 ▸; Yu *et al.*, 2019 ▸). A novel nanotwin has been investigated in nylon 6 nanorods with the twin axis aligned with the cylinder axis (Yu *et al.*, 2019 ▸). The 2D confined nanostructure of isotactic polypropylene exhibits five types of diversified α-phase crystals due to various types of molecular assembly of 3_1_ helices (Zeng *et al.*, 2019 ▸). The impact of triclinic symmetry on cylindrical confined hierarchical nanostructures has been illustrated by the rotation of the a*b* plane of the α-phase crystals in nylon 612 (Lai *et al.*, 2020 ▸).

The above symmetry studies on monoclinic or triclinic crystals under cylindrical constraints mainly illustrate the hierarchical nanostructures based on 2_1_ and 3_1_ helical chains assembling in confined spaces. However, a study of the tetragonal nanostructure assembling normally by 4_1_ or 7_2_ helices under 2D constraints has not yet been carried out. Furthermore, an interesting structural feature of tetragonal or similar cell symmetry is that, as found by Lotz and Lovinger, chain packing defects occur during the growth process (Ruan *et al.*, 2006*a* ▸; Lovinger *et al.*, 1993 ▸). For these reasons, in this study, we specifically selected poly(4-methyl-1-pentene) (P4MP1) (most occurring tetragonal crystals) as the model polymer to demonstrate the impact of tetragonal crystal symmetry on the 2D confined polymer nanostructure.

In addition, P4MP1 is an important engineering material with good performance and can be applied in medical appliances, labware materials and optical devices. Complex crystalline polymorphism can be found in the crystallization of P4MP1 (Griffith & Rånby, 1960 ▸; Danch & Gadomski, 1995 ▸; Lopez *et al.*, 1992 ▸; DeRosa *et al.*, 1995 ▸; Rånby *et al.*, 1962 ▸; Chen *et al.*, 2011 ▸). The crystal structure of P4MP1, phase I, had been solved by Tadokoro and co-workers using wide-angle X-ray diffraction (WAXD) (space group *P* 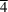 *b*2, *a* = 18.88 and *c* = 13.8 Å) (Kusanagi *et al.*, 1978 ▸; Ruan *et al.*, 2006*a* ▸). Four 7_2_ helical chains are packing in one tetragonal unit cell. The monoclinic crystal structure of phase II had been discovered by Takayanagi and co-workers, and was later refined by Lotz (*a* = 18.50, *b* = 10.43, *c* = 7.22 Å and γ = 113°) (Ruan *et al.*, 2006*b* ▸). Phase III was determined by De Rosa *et al.* (1994 ▸), in which 4_1_ chains pack in a tetragonal cell (*a* = 19.46 and *c* = 7.02 Å). The hexagonal structure of phase IV was first proposed by Charlet and Delmas, and was refined by De Rosa (*a* = 22.17 and *c* = 6.69 Å) (De Rosa, 1999 ▸; Aharoni *et al.*, 1981 ▸).

Here, P4MP1 nanorods crystallizing at a temperature of *T_c_* (100–220 °C) were made using the anodized alumina oxide (AAO) template. We first found an axially oriented crystal branching (phase I) of P4MP1 in the rod long axis under cylindrical confinement. The oriented crystal branching takes place along the rod long axis in 2D confinement and promotes 45°-tilted crystals to grow epitaxially from the parent crystals (*a*_1_-axis oriented crystals). The polymer nanostructure has been examined by 2D WAXD experiments. Detailed experiments on the morphologies, thermal analysis and crystal structure of the P4MP1 nanorods are presented below.

## 2. Results and discussion   

### 2.1. Morphologies   

**Figure 1:**
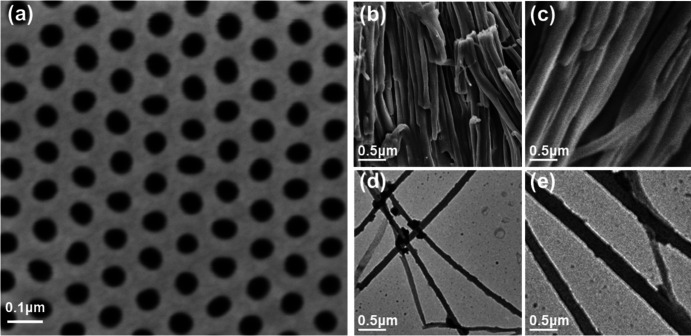
SEM images showing (*a*) a top-view of the anodic aluminium oxide (AAO) template with a pore diameter of 70 nm, and (*b*) the 70 and (*c*) the 300 nm rods of P4MP1. Transmission electron microscopy (TEM) bright field (BF) images of (*d*) the 70 and (*e*) the 300 nm rods of P4MP1.

The top-view SEM image of a nanoporous alumina template with self-organized cylindrical nanopores (70 nm) is shown in Fig. 1 ▸(*a*). The pore depth of the AAO template is around 120 µm. We used a 5–10% KOH solution to etch the templates to release the nanorods after isothermal crystallization at around 100 °C for 8 h. The released rods were then washed with distilled water several times and placed on a cover glass for the SEM test or on carbon-coated copper grids for TEM measurements.

The SEM images of the 70 and 300 nm rods of P4MP1 are shown in Figs. 1 ▸(*b*) and 1 ▸(*c*), respectively, while the TEM BF morphologies of the P4MP1 rods with average diameters of 70 and 300 nm are shown in Figs. 1 ▸(*d*) and 1 ▸(*e*), respectively. The SEM and TEM images both show that the lengths of the P4MP1 nanorods are in the range of tens of microns.

### 2.2. Multiple nucleation of P4MP1 nanorods under confinement   

**Figure 2:**
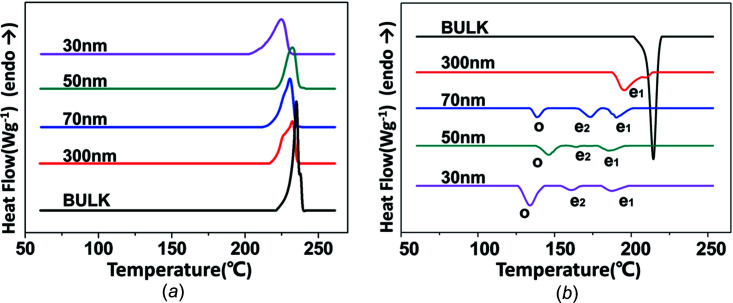
A set of DSC (*a*) heating and (*b*) cooling curves of the P4MP1 bulk and the 30, 50, 70 and 300 nm rods under 2D confinement (rate 10 °C min^−1^).

In differential scanning calorimetry (DSC) experiments, all the rod samples of P4MP1 are measured within the nanopores of the AAO template. A set of DSC heating curves for the bulk and for rods with different sizes (30, 50, 70 and 300 nm) is shown in Fig. 2 ▸(*a*). It can be seen that the melting peak broadens with decreasing rod diameter. Meanwhile, the onset shoulder temperature for an endothermic peak around 200 °C in the 30 nm rods decreases compared to that (220 °C) of the bulk, indicating that increased quantities of small crystallites are formed in strongly confined crystallization (small rods).

The DSC curves on cooling can reflect the nucleation type of crystals in 2D confinement (Lutkenhaus *et al.*, 2010 ▸; Duran *et al.*, 2011 ▸). The DSC cooling traces of PM4P1 in the bulk and confined rods with different sizes are shown in Fig. 2 ▸(*b*). One sharp and strong exothermic peak at 214 °C is displayed in the bulk upon cooling. Regarding the different pore sizes, there are obvious changes in the cooling curves of rods confined to nanoporous alumina. For the 300 nm rods, one broad exothermic peak at 195 °C is seen on the cooling runs. Compared with the cooling traces of the 300 nm rods, the cooling runs of the 70 nm rods display two exothermic peaks at 190 and 173 °C, and some additional exothermic processes arise at 138 °C. Similar observations are found in the 50 and 30 nm rods confined to nanoporous alumina. The two exothermic peaks at 190 and 173 °C of the 70 nm rods (indicated by ‘e_1_’ and ‘e_2_’ in Fig. 2 ▸ *b*) are assigned to heterogeneous nucleation. In addition, a homogeneous nucleation peak at 138 °C indicated by ‘o’) appears when the pore size is reduced to 70 nm. The homogeneous nucleation peak becomes stronger with decreasing rod size, reflecting the predominant heterogenous nucleation changing to dominant homogeneous nucleation as the degree of confinement increases (Duran *et al.*, 2011 ▸). The reason for this can be described as follows: according to the literature, in bulk crystallization, the crystallization is mainly nucleated by heterogeneous nucleation due to the existence of impurity (Duran *et al.*, 2011 ▸). With decreasing pore volume (rod size), the majority of the impurity is prohibited from entering into the nanopore. Therefore, homogeneous nucleation is more dominant in the system under strong confinement. On the other hand, the double heterogeneous nucleation peak reflects secondary and tertiary nucleation corresponding to nucleation at the smooth or corner surfaces, respectively. Epitaxial crystallization occurs in the second or tertiary nucleation in the high-temperature region.

### 2.3. 2D WAXD measurements for confined nanorods   

**Figure 3:**
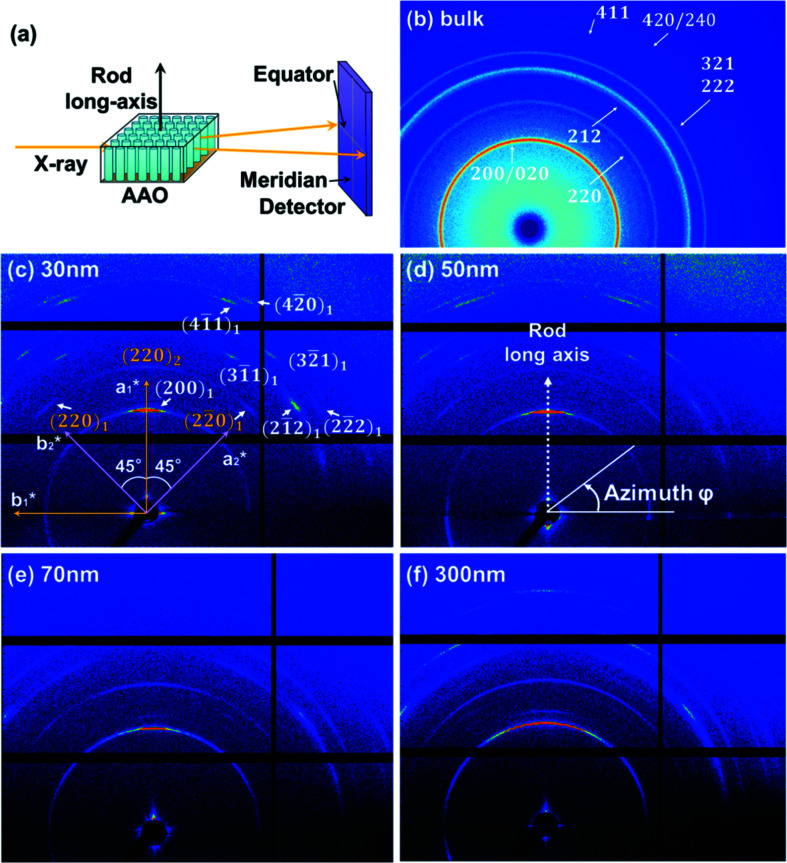
(*a*) An illustration of the perpendicular measurement geometry of the 2D WAXD experiments. The 2D WAXD patterns of (*b*) P4MP1 bulk and the (*c*) 30, (*d*) 50, (*e*) 70 and (*f*) 300 nm rods. The nanorods within the template were crystallized isothermally at *T_x_* = 100 °C for about 8 h before the WAXD experiments.

We utilized the Xeuss 2.0 instrument to measure the nanostructures of the 30, 50, 70 and 300 nm rods confined to the nanopores. The test geometry of the 2D WAXD experiments is depicted in Fig. 3 ▸(*a*).

Fig. 3 ▸(*a*) shows that the direction of the incident X-ray beam is normal to the rod long axis. The 2D WAXD patterns of the bulk and the 30, 50, 70 and 300 nm rods confined to the nanopores of the AAO template are shown in Figs. 3 ▸(*b*)–(*f*), respectively. Fig. 3 ▸(*b*) shows the ring pattern of the bulk, indicating that the crystals are randomly oriented. Compared to the ring pattern of the bulk, the 2D WAXD results for the nanorod samples (Figs. 3 ▸ *c*–*f*) show an anisotropic structure with respect to the rod long axis. The 2D WAXD results for the P4MP1 30 nm rods display several typical reflections at 2θ (Bragg angle) of 9.43, 13.35, 16.41, 16.77, 18.25, 20.59 and 21.18°, with corresponding *d*-spacings of 0.937, 0.662, 0.540, 0.528, 0.486, 0.431 and 0.419 nm, respectively (Fig. 3 ▸ *c*). Based on the phase I unit-cell parameters proposed by Tadokoro and co-workers (Kusanagi *et al.*, 1978 ▸), the reflections with *d*-spacings of 0.937, 0.662, 0.540, 0.528, 0.486, 0.431 and 0.419 nm can be assigned as 200/020, 220, 3
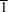
1, 2
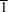
2, 3
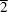
1/2
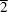
2, 4
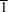
1 and 240/420, respectively. The observed diffraction Bragg angles, experimental and calculated *d-*spacings, and the Miller indices of the 2D WAXD patterns are listed in Table 1 ▸.

The *hk*0 diffractions (200, 020, 220 and 420) in Table 1 ▸ show that it is a [001]-zone X-ray diffraction pattern. The stronger meridian reflection with a *d*-spacing of 0.937 nm could be assigned as the 200 reflection. Therefore, the a* axis could be obtained from the 200 reflection, which is superimposed with the long axis of the rod (Fig. 3 ▸ *d*). According to the tetragonal crystal (α = β = γ = 90°), the angle between the a* and b* axes is 90°. The b* axis is thus perpendicular to the a* axis and is coincident with the equator. The equatorial reflections, such as the 020 reflections, were not observed due to the limit of the instrument. Similar results were observed in the 2D WAXD patterns of the 50, 70 and 300 nm rods (Figs. 3 ▸ *d*, 3*e* and 3*f*, respectively), which possesses the same *d*-spacings and reciprocal lattice.

**Figure 4:**
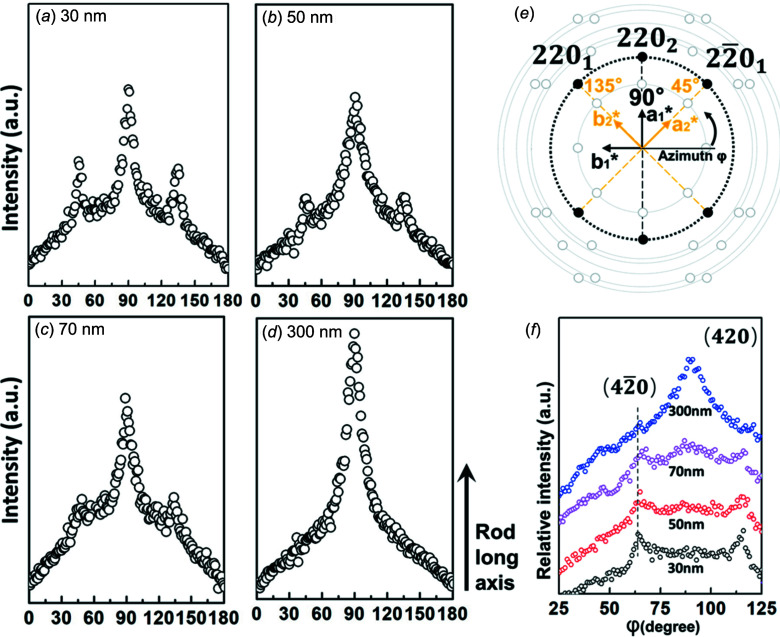
The azimuthal plots for the particular *d*-spacing *d* = 6.68 Å corresponding to the 220 diffractions of the 2D WAXD patterns (see Fig. 3 ▸) for the (*a*) 30, (*b*) 50, (*c*) 70 and (*d*) 300 nm rods. (*e*) In the 30, 50 and 70 nm nanorods, three 220 diffraction peaks appear at 45, 90 and 135°, respectively. (*f*) The azimuthal profiles for the 420 reflections of the 2D WAXD patterns of the 30 50, 70 and 300 nm rods.

Figs. 4 ▸(*a*), 4(*b*), 4(*c*) and 4(*d*) display the azimuthal plots of the 220 diffractions in the X-ray patterns (Figs. 3 ▸ *c*–*f*) of the 30, 50, 70 and 300 nm rods, respectively. Compared to the 220 reflections situated at 90° in weak confinement (300 nm rods; Fig. 4 ▸ *d*), three {220} planes can be clearly observed under strong confinement (rod size less than 100 nm; Figs. 4 ▸ *a*–*c*). The positions of the three {220} reflections correspond to the azimuthal scanning angles of 45, 90 and 135° in the reciprocal lattice (Fig. 4 ▸ *e*). One 220 diffraction (90°) situated at a meridian is coincident with the rod long axis, indicating that the 45°-tilted crystal has grown under confinement based on tetragonal geometry (Fig. 4 ▸ *e*).

The other two 220 or 2
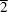
0 (45 or 135°) reflections are part of the [001]-zone diffraction pattern of the tetragonal crystals, reflecting that the *a*_1_-axis-oriented crystal co-exists in the P4MP1 nanorods. Under this circumstance, the *c* axes (chain axes) of the tetragonal crystals are perpendicular to the rod long axis and parallel to the direction of the X-ray beam.

On the other hand, it is also interesting to find that the azimuthal profiles of the 420 reflections in the different size rods (Fig. 4 ▸ *f*) show that one of the 420 peaks at 90° in the 300 nm rods (weak confinement) is split into two 420 peaks of the 30 nm rod (strong confinement). These two fractured 420 reflections of the 30 nm rods are part of the [001] zone diffraction pattern of the *a*_1_-axis-oriented crystals. Therefore, we can deduce that strong confinement promotes a more ordered structure of the *a*_1_-axis-oriented crystals.

**Figure 5:**
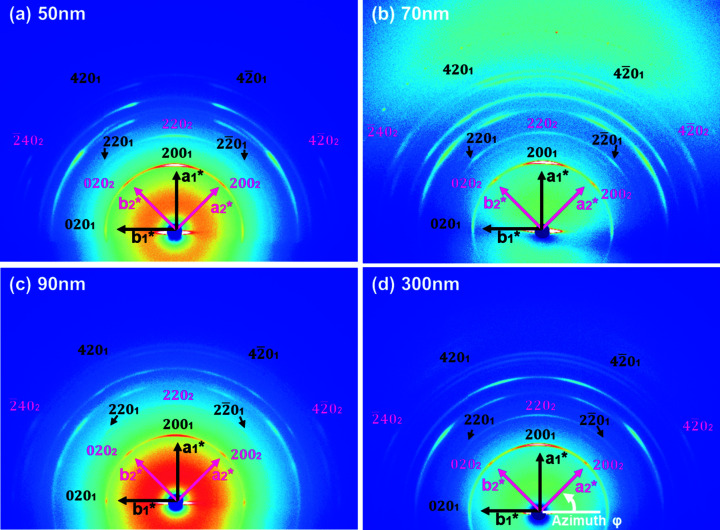
The 2D WAXD patterns of the (*a*) 50, (*b*) 70, (*c*) 90 and (*d*) 300 nm rods isothermally crystallized at 120 °C under atmospheric pressure for 48 h. The measurement geometry for the X-ray experiments is the same as that described in Fig. 3 ▸(*a*).

To obtain more orderly nanostructures to clearly show that this 45° tilting exists in all the rod sizes, the nanorod samples were crystallized isothermally at a higher temperature (120 °C) for a longer time (about 48 h) and we carried out X-ray experiments (Rigaku) on these rod samples with an identical test geometry to that shown in Fig. 3 ▸(*a*). Similar results are observed for the 50, 70, 90 and 300 nm rods. Surprisingly, the 200 and 020 reflections are clearly located at the geometric position of the [001]-zone diffraction pattern for the tetragonal crystals in reciprocal space. Figs. 5 ▸(*a*), 5(*b*), 5(*c*) and 5(*d*) show the 2D WAXD patterns of the P4MP1 nano­rods, crystallized isothermally at 120 °C for about 48 h, with diameters of 50, 70, 90 and 300 nm, respectively.

**Figure 6:**
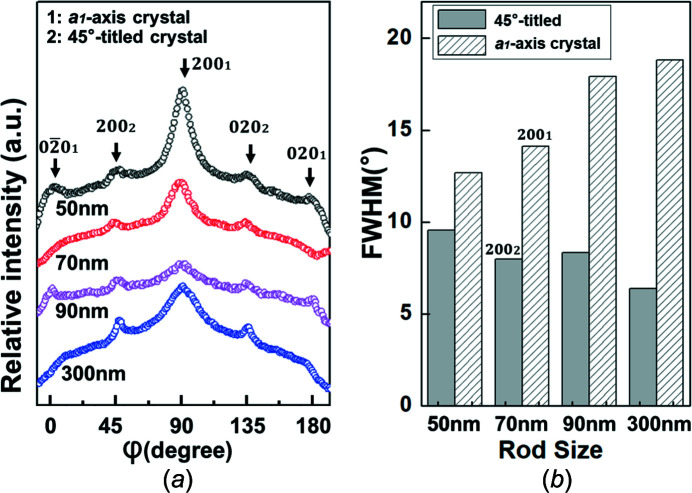
The azimuthal profiles for (*a*) the {200} reflections with *d* = 0.937 nm for the 2D WAXD patterns of the 50, 70, 90 and 300 nm rods. (*b*) The full width at half maxima (FWHM) of the 200 diffraction of the *a*_1_-axis-oriented and 45°-tilted crystals with different sizes.

The azimuthal scanning profiles of the 200 or 020 reflections with *d* = 0.937 nm for the X-ray patterns (Figs. 5 ▸ *a*–*d*) are shown in Fig. 6 ▸(*a*). Fig. 6 ▸(*a*) also displays that for all rod sizes, the 200 (φ = 90°) and 020 reflections (φ = 180°) belong to the *a*_1_-axis crystal, and the 200 (φ = 45°) and 020 (φ = 135°) reflections are attributed to the 45°-tilted crystal. Therefore, the 45°-tilted crystals obviously exist in all the rod sizes identified by the X-ray results (see Figs. 5 ▸ *a*–*d* and Fig. 6 ▸ *a*). Fig. 6 ▸(*b*) shows that the FWHM (full width at half maximum) value of the 200 reflections at 90° for the *a*_1_-axis crystal drops sharply when the rod size is less than 100 nm, and there is no obvious change in the FWHM values of the 200 reflections for the 45°-tilted crystal when reducing the size of the rods. Therefore, compared with the 45°-tilted crystals, the order of the molecular packing of the *a*_1_-axis crystal was more affected by the degree of confinement.

On the other hand, assuming that the defects of the *a*_1_-axis and 45°-tilted crystals are identical, the relative amount of the 45°-tilted crystals with respect to the rod size, *f*_45°/tilt_, can be estimated based on the azimuthal scanning profiles of the {200} reflections shown in Fig. 6 ▸(*a*). We calculate that the peak area ratio of the {200} diffractions belong to the 45°-tilted crystals, with φ = 45 and 135°, with respect to the total intensity of the {200} reflections. The amorphous halo is subtracted. The equations can be described as *f*_45°/tilt_ = [*I*(200)_45°_ + *I*(020)_135°_]/[*I*(200)_45°_ + *I*(020)_135°_ + *I*(200)_90°_ + *I*(020)_180°_], with *f*_45°/tilt_ for the 50, 70, 90 and 300 nm rods being about 19, 13, 24 and 16%, respectively, indicating that there is no significant change of *f*_45°/tilt_ with increasing degree of confinement.

Overall, on the basis of the above analysis, two kinds of tetragonal nanocrystal with respect to the rod long axis could be deduced: (i) an *a*_1_-axis-oriented crystal and (ii) a 45°-tilted crystal. This raises several questions, such as why do the *a*_1_-axis or 45°-tilted crystals co-exist under 2D confinement? How do the 45°-tilted crystals grow in confinement? How do molecular chains of P4MP1 arrange under 2D confinement?

**Figure 7:**
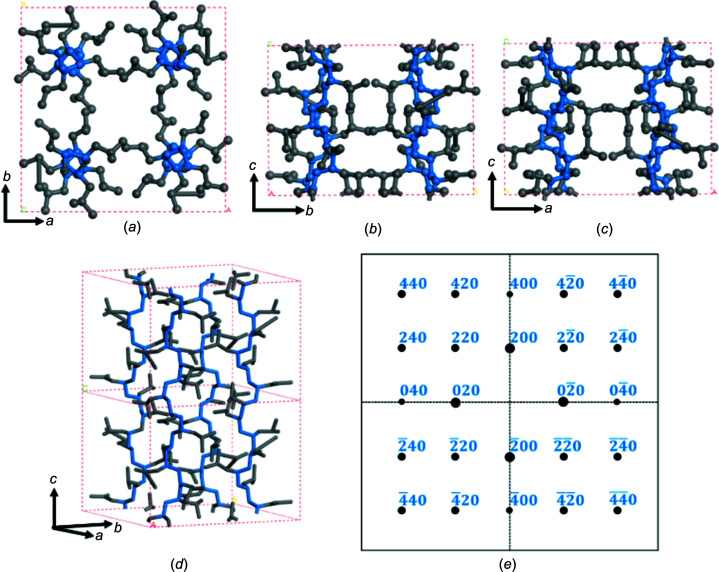
The (*a*) *ab*, (*b*) *bc* and (*c*) *ac* plane projections of phase I of P4MP1, as determined by Tadokoro and Lotz and co-workers. (*d*) The 3D structure of phase I of P4MP1. Gray sticks represent C atoms in the side chain and blue sticks represent C atoms in the main chain. (*e*) The simulated *hk*0 diffraction pattern of the P4MP1 crystal structure.

To understand these questions, we have built the molecular packing model of the phase I crystal with the help of the *Cerius* ^2^ modelling package using the structural data of Tadokoro and Lotz and co-workers (Kusanagi *et al.*, 1978 ▸; Ruan *et al.*, 2006*a* ▸). Figs. 7 ▸(*a*), 7(*b*) and 7(*c*) show the *ab*, *bc* and *ac* plane projections of the phase I crystal, respectively. A three-dimensional (3D) unit cell of the phase I crystal is shown in Fig. 7 ▸(*d*). Viewed from the *ab* projection shown in Fig. 7 ▸(*a*), four 7_2_ chains of P4MP1 are located in one tetragonal cell. Figs. 7 ▸(*b*)–(*d*) show that the chain axis of P4MP1 is along the *c* axis. According to the packing model of phase I, we can obtain the [001]-zone simulated pattern of phase I crystals (Fig. 7 ▸ *e*). Therefore, our experimentally observed *hk*0 diffractions (200, 020, 220 and 420) are consistent with the [001]-zone calculated results.

### 2.4. *a*_1_-axis-oriented crystals   

**Figure 8:**
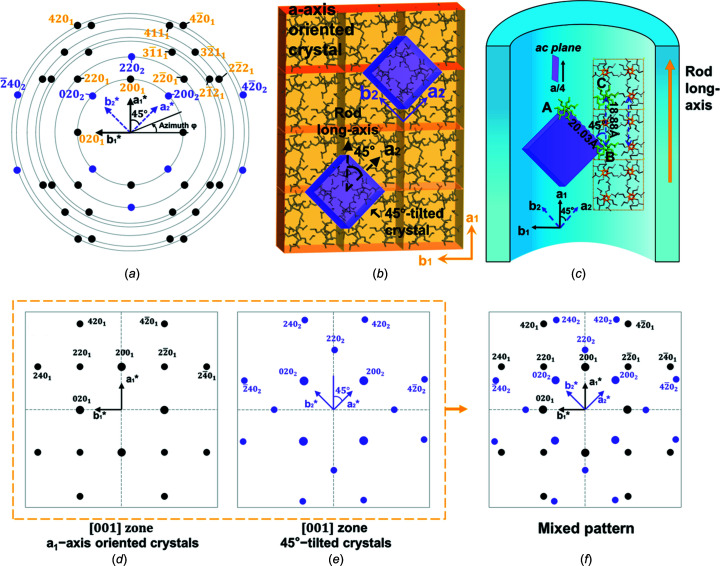
(*a*) Diffraction pattern of the phase I crystals in 2D confinement extracted from the experimental results (Figs. 3 ▸ and 5 ▸). (*b*) A schematic illustration of the cell orientation in the parent crystal (marked in orange) and the 45°-tilted crystals (purple). (*c*) The probable branching mechanism between the *a*_1_-axis-oriented and 45°-tilted crystals. The orange arrow represents the rod long axis. The helix *A* and two neighbouring helices *B* and *C* are marked in green in part (*c*). (*d*) The calculated diffraction pattern with the [001]-zone for the *a*_1_-axis-oriented crystals. (*e*) The calculated diffraction pattern for the 45°-tilted crystals; the [001]-zone diffraction pattern is rotated 45° clockwise in the *ab* plane around the *c* axis. (*f*) The diffraction pattern of the superposition of parts (*d*) and (*e*).

Fig. 8 ▸(*a*) displays a typical 2D WAXD pattern of the P4MP1 nanorods. As mentioned above, the *a*_1_*-axis orientation can be inferred from the meridional 200 diffraction. The *a*_1_ axis is superimposed on the *a*_1_* axis and is consistent with the rod long axis. The b* axis can be deduced with reference to the equatorial 020 reflections. Combining the 45°(φ)- and 135°-{220} reflections, a preferential [200]-directed crystal in the rod long axis was defined as the *a*_1_-axis-oriented crystal. The reason for the occurrence of *a*_1_-axis-oriented crystals in the nanopore can be described as follows: in strong confinement, only a lamellar with a specific <*hk*0> orientation could develop (Steinhart *et al.*, 2006 ▸). Meanwhile, it has been found that the fast growth direction of fibrils in the P4MP1 bulk spherulite is along the *a*_1_-axis direction. In 2D confinement, the [200] direction of fibril crystals tends to be coincident with the rod long axis. Therefore, the occurrence of the *a*_1_-axis-oriented crystal results from the collaborative growth of hierarchical assembling in P4MP1 under cylindrical con­straints.

### 2.5. 45°-tilted crystals   

A schematic illustration of the *a*_1_-axis-oriented and 45°-tilted crystals is shown in Fig. 8 ▸(*b*). As analyzed above, the molecular chain axis of the 45°-tilted and *a*_1_-axis-oriented crystals is parallel to the direction of the X-ray beam, that is, perpendicular to the rod long axis. The *a*_2_ axis of the 45°-tilted crystals is rotated 45° clockwise away from the *a*_1_ axis. A similar observation was made in solution-grown crystals of P4MP1, where unusual 37° epitaxial crystal branching daughter crystals were discovered by Lotz and co-workers (Ruan *et al.*, 2006*a* ▸). Based on microscopy studies of the daughter and mother single crystals of P4MP1, we deduce that the origin of the 45° angle between these two types of crystals in 2D confinement probably results from an epitaxial crystal branching occurring in the *a*_1_-axis-oriented and 45°-tilted crystals.

An illustration of the epitaxial branching mechanism between the *a*_1_-axis-oriented and 45°-tilted crystals is shown in Fig. 8 ▸(*c*). The nucleation takes place on the *ac* layer of the *a*_1_-axis-oriented crystal and helix *A* (marked in green) slips by 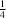 *a*. The *AB* distance (two neighbouring helices *B* and *A* in the new crystal; Fig. 8 ▸ *c*) is about 20.03 Å and the *BC* distance (two neighboring helices *B* and *C* of the parent crystal; Fig. 8 ▸ *c*) is 18.88 Å. The size difference of the two lattices is (20.03 − 18.88)/18.88 ≃ 6% and falls within the reasonable range of lattice epitaxy. This indicates that the 45°-tilted crystals probably grow epitaxially from the *a*_1_-axis-oriented crystals. There are several cases of a similar shift occurring in syndiotactic polystyrene and polypropylene (Tosaka *et al.*, 1998 ▸, 1999 ▸; Lotz *et al.*, 1988 ▸; Lovinger *et al.*, 1993 ▸). This molecular shift and nucleation are a typical structure defect in the tetragonal unit cell of polymers.

On the other hand, with the help of the *Cerius^2^* modelling package, the [001]-zone diffraction pattern of the *a*_1_-axis-oriented and 45°-tilted crystals can be calculated, as shown in Figs. 8 ▸(*d*) and 8(*e*), respectively. The diffraction pattern in Fig. 8 ▸(*e*) can be obtained by clockwise rotation of the [001]-zone diffraction pattern of the *a*_1_-axis-oriented crystals (Fig. 8 ▸ *d*) by 45° in the *ab* plane around the *c* axis. The superimposed diffraction patterns (Fig. 8 ▸ *f*) show a symmetric and mixed pattern, which resembles the 2D WAXD experimental results of the P4MP1 nanorods shown in Fig. 8 ▸(*a*). Therefore, the simulation results shown in Fig. 8 ▸(*f*) confirm the coherent crystal branching of the tetragonal nanostructure under cylindrical confinement.

In addition, the unique 2D confined tetragonal nano­structure is significantly different from the X-ray fiber structure or the bulk crystallization. For the X-ray fiber diagram of the phase I crystals in P4MP1, the *c* axis (chain axis) is parallel to the fiber axis, and the *hk*0 reflections 200, 220, 420, *etc*., are located at the equator, while the 113 reflections are on the meridian (Kaji *et al.*, 1974 ▸). In our case, the 200, 220, 420, *etc*., reflections correspond to the reciprocal spatial distribution of the [001]-zone diffraction pattern of the phase I crystal. It is known that crystals are randomly oriented in the bulk crystallization of P4MP1. Compared with the arbitrary distribution of lamellar crystals grown from solution, tetragonal crystals in 2D confinement are controlled to form an ordered structure. However, the parent and daughter crystals in solution cannot assemble an ordered structure on a large length scale.

## 3. Conclusion   

P4MP1 rods with different sizes under various degrees of confinement exhibit similar nanostructures. A detailed structure analysis based on WAXD, DSC and SEM experiments indicates that tetragonal crystals (phase I) are formed in rods with diameters of 30, 50, 70 and 300 nm. Compared to the broad diffraction arcs of the 2D WAXD pattern of the 300 nm rod (weak confinement), the 2D X-ray diffractions of the 30 nm rod change sharply, implying that the molecular chains and lamellae assemble in a more orderly manner under strong confinement due to a collaborative hierarchical assembly of P4MP1 in 2D confinement.

The impact of tetragonal symmetry on the co-operative assembly of 2D confined nanostructures of P4MP1 has been investigated. We first found a coherent 45° crystal branching of the anisotropic tetragonal nanostructure manipulated by cylindrical confinement. Two sets of [001]-zone diffraction patterns of phase I crystals with 45° in the *ab* plane are superimposed on the 2D WAXD patterns of the P4MP1 nanorods. The 200 reflection located at 45 and 90° (meridian) on the azimuthal scan profiles with a *d*-spacing of 0.937 nm reflected the formation of 45°-tilted and *a*_1_-axis-oriented crystals, respectively. The formation mechanism of the *a*_1_-axis-oriented crystals is governed by the fastest crystal growth direction (the [200] direction), coincident with the rod long axis. More importantly, the 45° angle between the *a*_1_-axis-oriented and branched crystals indicates that a tetragonal structural defect (*ac* layer shift) takes place along the rod long axis, which helps the new crystals to grow epitaxially from the *a*_1_-axis-oriented crystals in 2D confinement and results in a coherent crystal branching under cylindrical confinement. In principle, our research on the chain assembly of tetragonal crystals under confinement could contribute to the understanding of the impact of tetragonal symmetry on molecular chain self-assembly in polymer nanostructures under confinement.

## 4. Experimental   

### 4.1. Sample and materials   

Two low and high molecular weight (MW) P4MP1 samples were obtained commercially from Sigma–Aldrich and used as received. For the low-MW sample, based on the ASTM D1238-01 standard, the melt flow index is 180 g/10 min (260 °C/5 kg) and the density is around 0.833 g/ml (25 °C). The melt index of the high-MW sample is 26 g/10 min (260 °C/5 kg) and the density is 0.835 g/ml (25 °C). The AAO templates were purchased from Shangmu Technology Co. Ltd. The average pore diameters of the AAO template are 30, 50, 70, 90 and 300 nm, and the pore depth of the AAO template is about 150 µm.

The cylindrically confined crystallization of P4MP1 was achieved by infiltrating the P4MP1 melt into the nanopores of the AAO template at a temperature of 260 °C. The residual bulk on the surface of the AAO template needs to be removed. In this article, both high- and low-MW samples provide the same phase behaviour and nano­structure at atmospheric pressure. Both samples pro­vided tetragonal phase I during crystallization within a crystallization temperature range of 100–220 °C. Similar 2D WAXD results for the bulk and nanorod samples can be observed for high- and low-MW commercial samples, indicating that this coherent crystal branching is an impact of common tetragonal symmetry on the nanostructure of P4MP1 in 2D confinement. Therefore, there is no need to identify specific samples in this report.

### 4.2. Differential scanning calorimetry (DSC)   

All thermal analyses were operated under dry nitrogen with a PerkinElmer DSC 8500 instrument. The samples were first heated to the equilibrium melting temperature and subsequently cooled to 25 °C. The samples were then heated to the equilibrium melting temperature again. The second heating and first cooling curves were recorded. The heating and cooling rates are both 10 °C min^−1^. The weights of all the pans were within a deviation of 0.002 mg.

### 4.3. Scanning electron microscopy (SEM)   

The morphologies of the P4MP1 nanorods with different diameters were captured by SEM (Hitachi SU-70). The P4MP1 nanorods were placed on a glass film and coated with gold before measurements.

### 4.4. Transmission electron microscopy (TEM)   

A JEOL-1100 instrument was used to obtain the BF image of the P4MP1 nanorods, with an acceleration voltage of 110 kV. The P4MP1 nanorod samples were released from the AAO template etched by 5% aqueous KOH solution. P4MP1 nanorods in a distilled water droplet were then cast onto the carbon-coated copper grids and were dried in air.

### 4.5. Wide-angle X-ray diffraction (WAXD)   

The 2D WAXD experiments (Fig. 3 ▸) of the nanorods within the AAO template were carried out using a Xeuss 2.0 wide-angle X-ray diffraction system under a 40 kV working voltage and a 30 mA current. The Xeuss 2.0 instrument from French Xenocs was equipped with a Pilatus3 detector and a MetalJet-D2 X-ray source (λ = 0.134 nm). The 2D WAXD experiments (Fig. 5 ▸) were carried out using a Rigaku wide-angle X-ray diffraction system equipped with a Hypix-6000 detector and an FR-X rotating-anode X-ray source (λ = 0.154 nm).

## Figures and Tables

**Table 1:** Observed diffraction Bragg angles, experimental and calculated *d*-spacings and Miller indices of the 2D WAXD patterns 2θ_obs_ is the observed diffraction Bragg angle. *d*_obs_ is the observed *d*-spacing, corresponding to the WAXD patterns shown in Figs. 3 ▸(*c*)–(*f*). *d*_cal_ is the calculated *d*-spacing using the unit-cell parameters *a* = *b* = 1.888 and *c* = 1.38 nm proposed by Tadokoro and Lotz (Kusanagi *et al.*, 1978 ▸; Ruan *et al.*, 2006*a* ▸).

2θ_obs_ (°)	*h*	*k*	*l*	*d*_obs_ (nm)	*d*_cal_ (nm)
9.43	2	0	0	0.937	0.944
	0	2	0	0.937	0.944
13.35	2	2	0	0.662	0.668
16.41	3	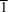	1	0.540	0.548
16.77	2	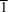	2	0.528	0.534
18.25	3	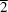	1	0.486	0.490
18.25	2	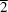	2	0.486	0.479
20.59	4	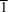	1	0.431	0.435
21.18	4	2	0	0.419	0.422
	4	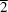	0	0.419	0.422
	2	4	0	0.419	0.422
